# Parsing GTF and FASTA files using the eccLib Library

**DOI:** 10.1093/bioinformatics/btaf558

**Published:** 2025-10-07

**Authors:** Tomasz Chady, Zuzanna Karolina Filutowska

**Affiliations:** Department of Mathematical Statistics and Data Analysis, Faculty of Mathematics and Computer Science, Adam Mickiewicz University, ul. Uniwersytetu Poznańskiego 4, Poznań, 61-614, Poland; Department of Gene Expression, Faculty of Biology, Adam Mickiewicz University, ul. Uniwersytetu Poznańskiego 6, Poznań, Wielkopolska, 61-614, Poland

## Abstract

**Summary:**

Leveraging the Python/C API, eccLib was developed as a high-performance library designed for parsing genomic files and analysing genomic contexts. To the best of the authors’ knowledge, it is the fastest Python-based solution available. With eccLib, users can efficiently parse GTF/GFFv3 and FASTA files and utilize the provided methods for additional analysis.

**Availability and implementation:**

This library is implemented in C and distributed under the GPL-3.0 licence. It is compatible with any system that has the Python interpreter (CPython) installed. The use of C enables numerous optimizations at both the implementation and algorithmic levels, which are either unachievable or impractical in Python.

## 1 Introduction

GTF/GFF version 2 and FASTA file formats are widely used in genomics owing to their simplicity and ease of use. Consequently, parsing these files is often the first step in many projects, and can become a bottleneck. Indeed, this was the experience of the authors, who found a Python-based parser to be too slow and memory-intensive for their requirements. To address this issue, numerous libraries exist to facilitate this process. The gffutils module (Dale 2011) parses GTF files into an SQLite database. The gtfparse module (Openvax 2015), the Biopython-affiliated bcbio-gff module (chapmanb 2013), and the GTF parser within the PyRanges library (Stovner and Sætrom 2020) all return representations of GTF files. For FASTA files, the Biopython library includes a parser called SeqIO, the C-based pyfastx (Du *et al.* 2021) provides both FASTA and FASTQ parsing, while the standalone libraries fastaparser (Kronopt 2020) and fastapy (Zielezinski *et al.* 2022) also support FASTA parsing.

However, the performance of some of these libraries may be suboptimal. To overcome these limitations, eccLib, a performance-oriented library written in C for Python was developed. The primary objective of eccLib is to accelerate the parsing of GTF and FASTA files in Python while providing efficient data structures for genomic data. Users can expect significant performance improvements, particularly with large datasets.

## 2 Methods

The standard implementation of the Python interpreter, CPython, provides an interface for integrating C code, known as the Python/C API. Using this API, developers can write C code that, once compiled and linked to the Python interpreter, can be invoked directly from Python code. This is exactly the interface utilized by numpy (Harris *et al.* 2020) to deliver excellent performance for numerical computations. Development of eccLib was not without its challenges owing to its low-level nature. Debugging was particularly problematic when C memory errors propagated into Python reference counting errors. Despite this, the performance gains—even from a direct port of existing Python code into C—can be significant.

However, the true performance improvements arise from low-level access to memory, which permits optimizations that are either unachievable or impractical in Python. Within eccLib, this low-level access was utilized to optimize the parsing of GTF and FASTA files, primarily by reducing the number of memory allocations to a minimum and avoiding unnecessary data copying, which is a common issue in high-level languages such as Python.

Numerous C libraries for parsing FASTA files already exist, such as seqtk (Li 2012) and SeqAn3 (Reinert *et al.* 2017). The authors deliberately avoided reliance on third-party libraries, choosing instead to implement their own FASTA parser. The use of existing libraries would entail substantial processing overhead, owing to the translation of native C types into Python objects, a step circumvented in eccLib by writing directly into Python objects.

### 2.1 Data types

Access to the internals of output data structures is crucial for optimizing performance. To that end, several custom data types were created. The GtfDict class is a Mapping object used to store data from GTF files. The core GTF entry fields are stored as separate members within the object, permitting access without a hash map lookup. For storing GTF attributes, a custom C hash map is employed, which is faster than a Python dict. This hash map, from the hashmap.h library (Warden *et al.* 2010), utilizes the xxHash hashing function (Collet 2012), thereby improving overall performance. Additionally, the GtfDict class includes several methods for basic data analysis, such as calculating the overlap between two GTF entries, querying sequence length, and checking if one sequence is contained within another.

The FastaBuff class is used to store FASTA DNA data. The set *I* of IUPAC nucleotide codes (Cornish-Bowden 1985), can be mapped to a power set of DNA bases using f:I→P({A,C,T,G}). In such a mapping, f(N)={A,C,T,G} and f(D)={A,G,T}. This power set can then be mapped to a 4-bit integer, where each bit represents the presence of a specific base, using $g:P(\{A,C,T,G\})\to {\left\{0,1\right\}}^{4}$. The composition of these two functions allows for the efficient storage of sequences as compact binary data, specifically as unsigned 4-bit integers. In this implementation, these 4-bit integers are packed into bytes, enabling a memory usage reduction of at least 50% compared to Python str objects, with minimal performance overhead. For instance, the sequence ‘CG’ may be represented as ${\left(01000010\right)}_{2}$, and the sequence ‘DNT’ as ${\left(10111111\;00010000\right)}_{2}$. For non-nucleotide characters, a Python str object is employed as a fallback.

### 2.2 GTF parsing

The principal challenge in GTF parsing lies in string tokenization and the optimal storage of attributes. To address this, a custom C tokenizer that does not modify the input data is utilized—thereby avoiding additional memory allocations—and a third-party hash map library is employed for caching and attribute storage. Caches are utilized for storing already encountered keys and values during parsing, thus avoiding redundant memory allocations for identical strings. In addition, the process of resolving URL and UTF-8 encodings was optimized by using a custom C function that simultaneously resolves both encodings, which significantly increases overall performance. Two parser styles are available: a single-pass parser that loads the entire file into memory and an iterative parser that sequentially streams the file and processes the data.

### 2.3 FASTA parsing

Parsing the FASTA format is simpler than GTF processing, with the main challenge being sequence storage. To address this, the FastaBuff class is used by the primary parser parseFASTA(), reducing memory usage compared with Python bytes or str objects at the cost of a slight performance overhead. For non-nucleotide sequences, parsing into str objects remains available. Unlike pyfastx, which indexes external files and loads sequences lazily, eccLib stores data in memory buffers. Although less memory-efficient, this design is more robust and flexible, enabling support for Python io streams. The authors prioritize usability over memory efficiency in this trade-off. As with the GTF parser, both single-pass and iterative variants are implemented.

## 3 Results

To assess the performance of the eccLib library, a benchmark was conducted. This benchmark was performed on a Linux system equipped with an Intel i9-9900KF CPU, 48.0 GB of RAM, and an SSD. The objective was to measure how quickly each parser could return a representation of the provided file. For iterative parsers, the outputs were appended to a list, thereby simulating the behaviour of a non-iterative parser.

In turn, 40 new Python instances were launched for each parser, measuring the time with the datetime module and memory usage with the GNU Time utility. To better illustrate overall performance, the Time-Memory Product (TMP) was calculated using the formula TMP = Time × Memory, expressed in seconds × gigabytes.

Methods that have achieved the best result in at least one column are summarised in Table 1.

### 3.1 GTF parsing benchmark

**Figure 1.**
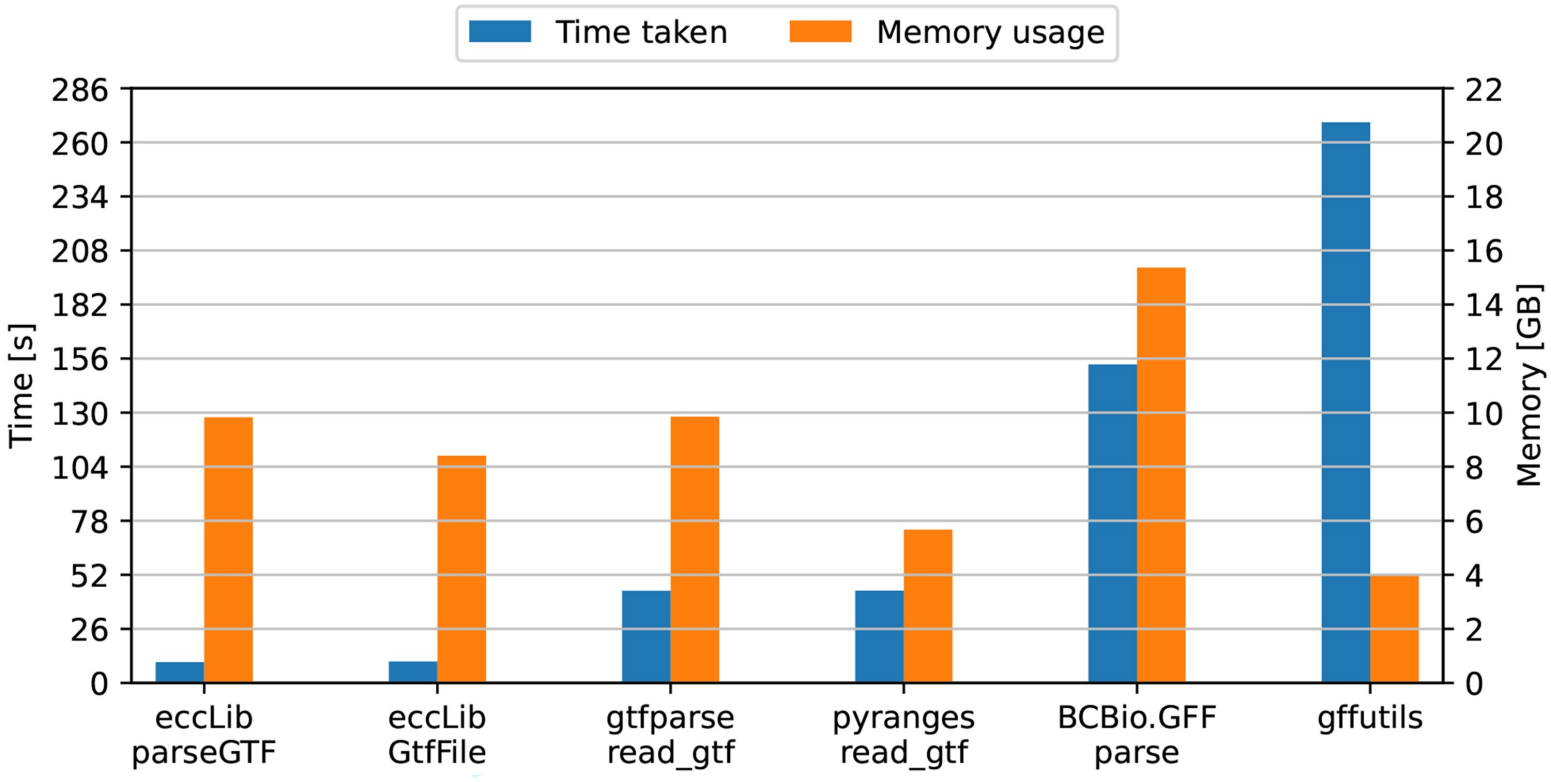
Speed and peak memory usage of different GTF parsing methods. The methods presented here, GtfFile and parseGTF, achieved TMP scores of 86.70 and 98.24, respectively. Third-party methods, pyranges.read_gtf, gtfparse.read_gtf, gffutils, and BCBio.GFF.parse achieved TMP scores of 251.76, 436.73, 1086.16, and 2353.70, respectively.

Parsing methods within eccLib were compared against one another, the gtfparseread_gtf function, BCBio.GFF.parse, and the PyRanges read_gtf function. The benchmark file used for speed tests was the 1.4 GB Homo_sapiens.GRCh38.108.gtf from Ensembl (Dyer *et al.* 2025), chosen for its size and accessibility. The results are presented in Fig. 1.

The results indicate that methods presented here are significantly faster at parsing GTF files compared to the alternatives, albeit with higher memory usage. eccLib.GtfFile parser is 4.31 times faster than pyranges.read_gtf, while consuming 1.48 times more memory.

Contrary to expectations, the iterative parser was not significantly slower than the non-iterative parser. The authors attribute this to the hardware characteristics, which mitigated the performance penalty incurred by additional I/O operations.

One factor that may have influenced the results is the high number of repeated GTF attribute keys and values in the benchmark file; however, the authors do not believe this had a significant impact on the overall outcomes.

### 3.2 FASTA benchmark

**Figure 2.**
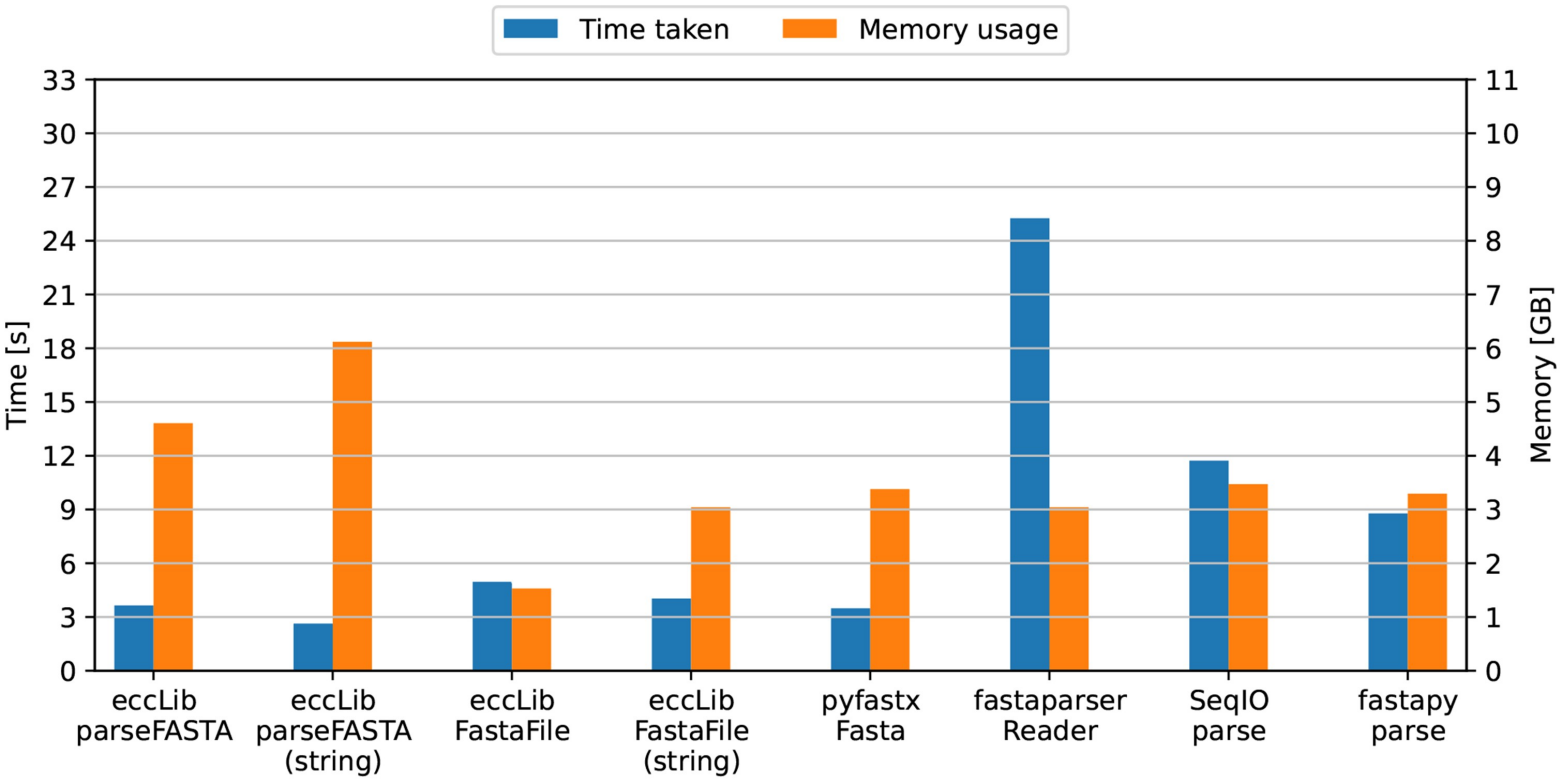
Speed and peak memory usage of different genomic FASTA parsing methods. Methods from eccLib were benchmarked parsing both into FastaBuff and str. The best overall performance was achieved by eccLib.FastaFile iterative parser with TMP score of 7.59, followed closely by pyfastx.Fasta with score of 11.78 and eccLib.FastaFile in string mode with score of 12.26. The single-pass parser eccLib.parseFASTA yielded a TMP score of 16.80 and 16.26 in string mode. fastapy.parse, SeqIO.parse, and fastaparser.Reader achieved scores of 28.93, 40.71, and 76.83, respectively.

Testing indicates that the parseFASTA() function in eccLib outperforms contemporary FASTA parsers. The benchmarking was conducted using the same methodology as the GTF parsing benchmark. The test file used was the 3.2 GB Homo_sapiens.GRCh38.dna.primary_assembly.fa from Ensembl. The averaged results are shown in Fig. 2.

While eccLib.parseFASTA proved to be the fastest when parsing into a str object, it incurred higher memory usage. The most efficient memory usage was achieved by eccLib.FastaFile employing FastaBuff, parsing a 3.2 GB file while using only 1.6 GB of memory.

**Figure 3.**
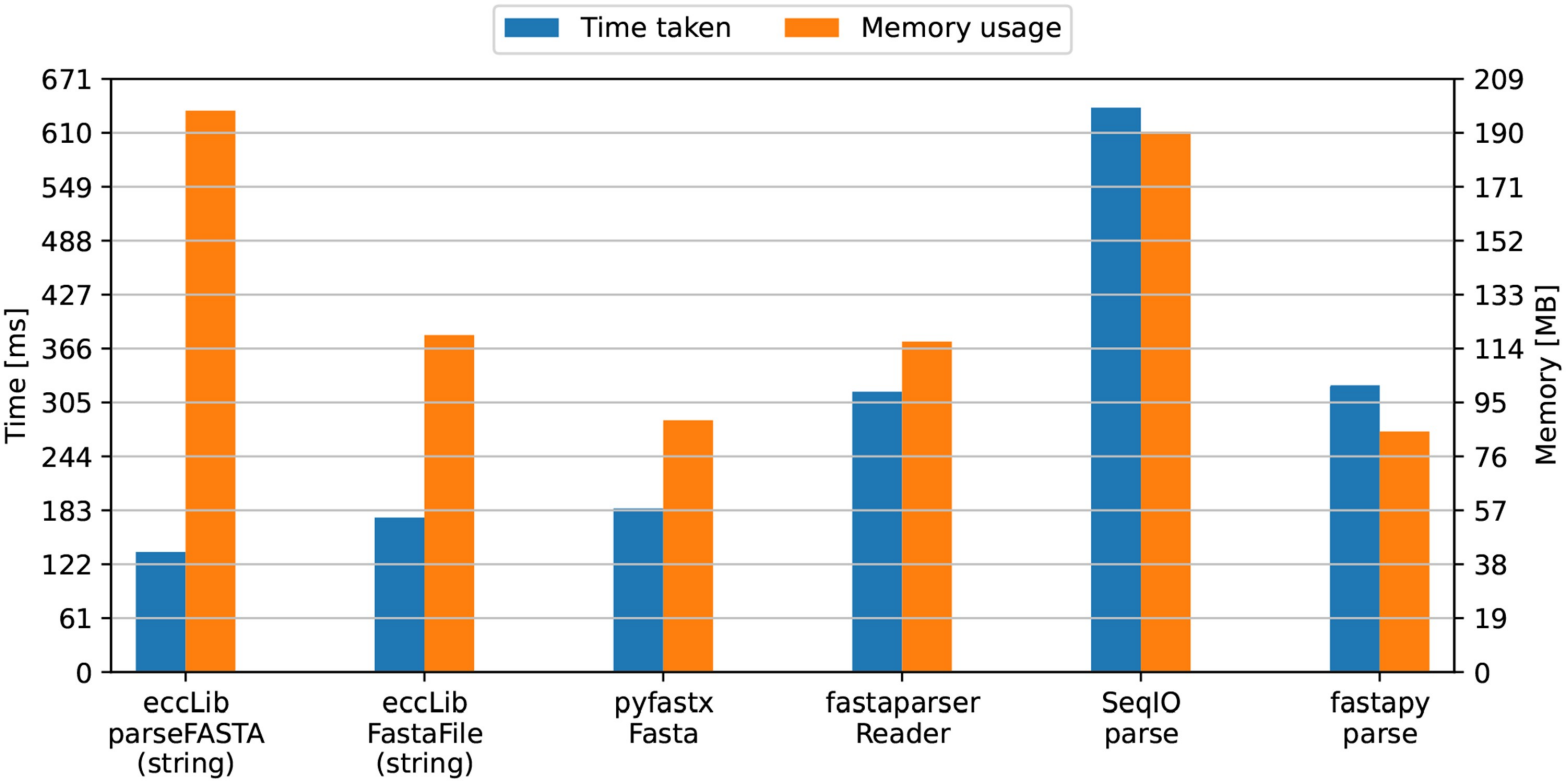
Speed and peak memory usage of different protein FASTA parsing methods. pyfastx.Fasta achieved the best TMP of 0.01643, followed by eccLib.FastaFile with TMP of 0.02075 and eccLib.parseFASTA with TMP of 0.02687. fastapy.parse, fastaparser.Reader, and SeqIO.parse achieved TMP scores of 0.02750, 0.03693, and 0.01216, respectively.

Since eccLib handles DNA sequences differently from protein sequences, a separate benchmark was conducted using the 85.1 MB Homo_sapiens.GRCh38.pep.all.fa from Ensembl. The results are illustrated in Fig. 3.

Here, the relative performance of eccLib is notably poorer; however, the speed advantage remains; the increased memory usage is largely attributable to the fact that protein sequences are not stored as efficiently as genomic sequences.

In order to ensure a fair comparison, the pyfastx. Fasta parser was forced to load the sequences into memory. Since pyfastx lazily loads sequences into memory, comparing pyfastx to other parsers that immediately load sequences into memory is meaningless without such a measure. Alongside this measure, the fastaparser.Reader was used in ‘quick’ mode to manage memory usage.

### 3.3 Comparison to native C/C++ parsers

**Figure 4.**
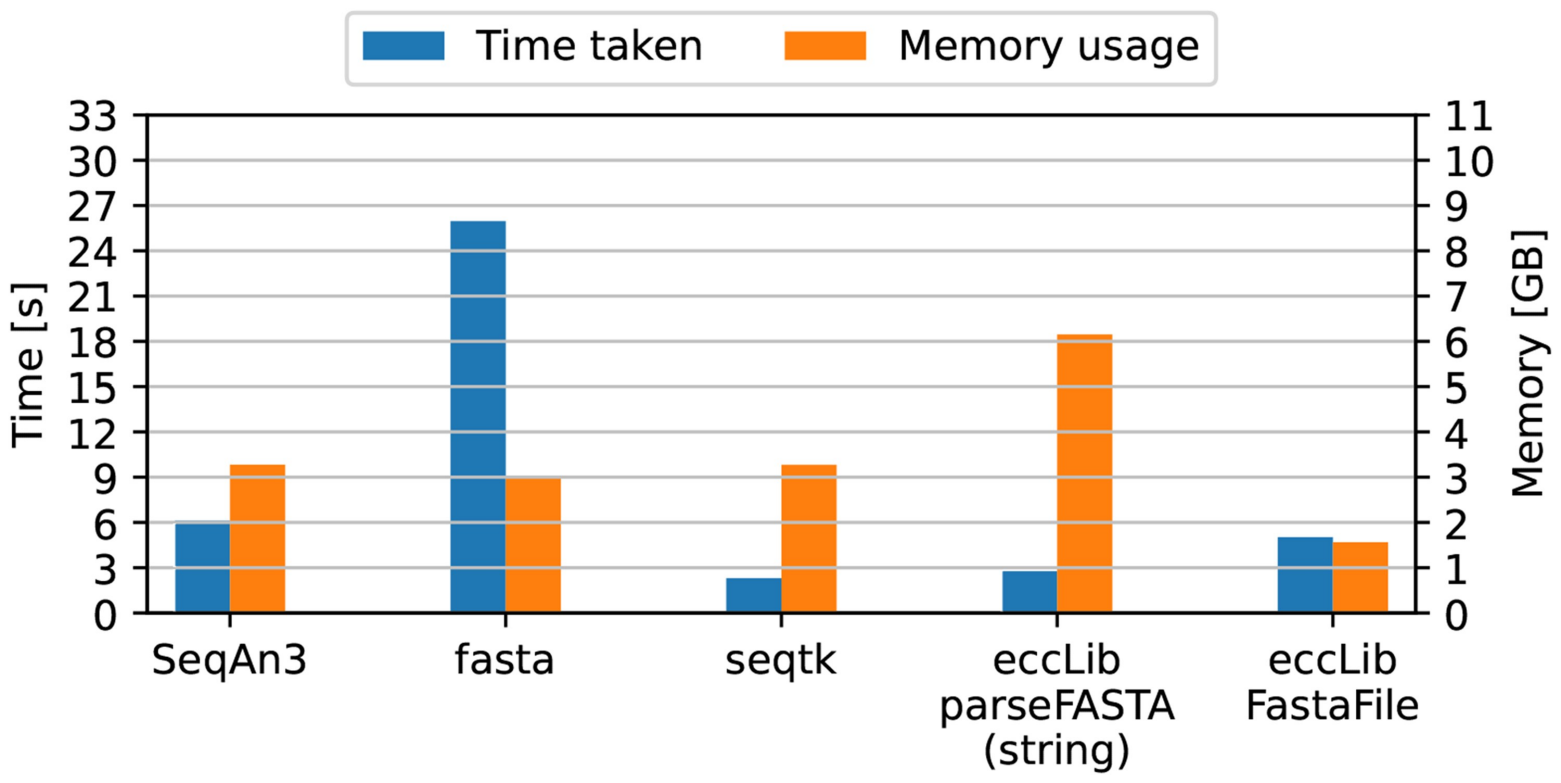
Comparison of eccLib FASTA parsing methods and native C/C++ parsers. The best performance was achieved by seqtk library with TMP of 7.54, followed by eccLib.FastaFile with 7.84. eccLib.parseFASTA, SeqAn3, and fasta achieved TMP scores of 17.03, 20.03, and 78.63, respectively.

**Figure 5.**
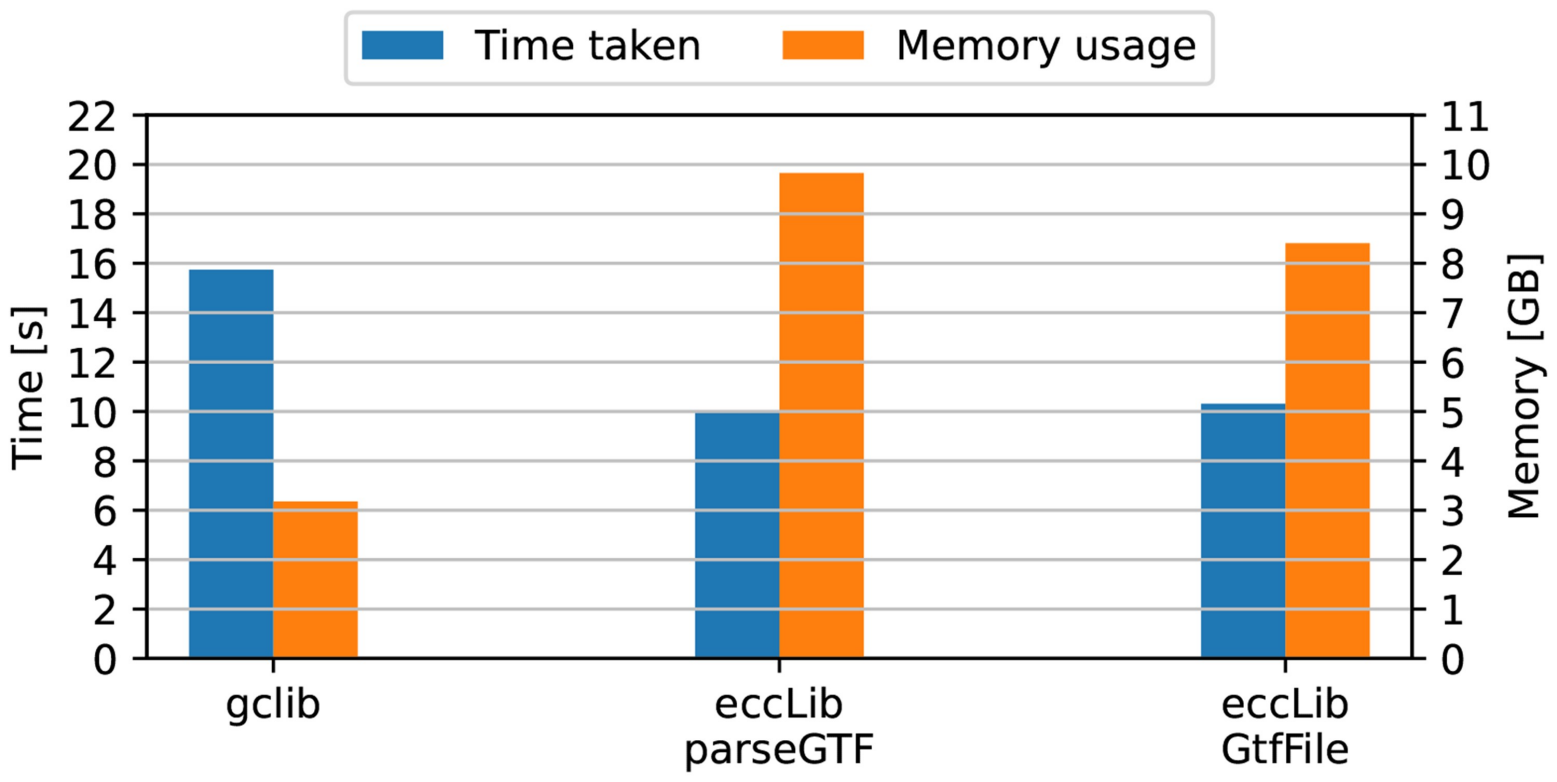
Comparison of eccLib GTF parsing methods and native C/C++ parsers. The best performance was achieved by GCLib, with TMP of 50.05. This is owing to its efficient memory usage despite its less-than-optimal speed. eccLib.GtfFile and eccLib.parseGTF achieved TMP scores of 101.86 and 115.04, respectively.

An additional benchmark was done, with eccLib parsers being compared to native C/C++ libraries, such as seqtk (Li 2012), fasta (Brunet 2017), GCLib (Pertea *et al.* 2015), and SeqAn3 (Reinert *et al.* 2017). Identical methodology to previous benchmarks was applied, except native C/C++ parsers were utilized in standalone executables, while eccLib parsers were used in Python scripts. Results of the FASTA benchmark are shown in Fig. 4, and GTF benchmark in Fig. 5.

Despite the significant overhead imposed by the Python C/API, eccLib performs admirably when compared to native C/C++ parsers, with the parseFASTA function in string mode achieving near identical speed to seqtk, and outperforming SeqAn3. It’s worth noting, that htslib (Bonfield *et al.* 2021) uses the seqtk library for parsing FASTA files.

The benchmarks indicate that eccLib is faster than GCLib, while having significantly worse memory usage. This is due to extensive use of memory aliasing and shared ownership in GCLib, which would be impractical in a Python environment. GCLib was forced to parse all GTF entries, which is not done by default, in order to ensure a fair comparison. Unfortunately, as of the writing of this article, SeqAn3 does not support GTF parsing, despite SeqAn2 supporting it.

## 4 Discussion

The eccLib library provides a high-performance solution for parsing GTF and FASTA files in Python, thereby significantly enhancing project performance. eccLib was built with the Python/C API in mind, allowing for performance closely matching that of native C parsers, which would not be feasible if implemented directly in Python, or if it were to rely on a pre-existing C library.

Although it utilizes more memory while parsing GTF files than alternative approaches, this is a consequence of its adoption of hash maps rather than fixed-field tables, which better accommodate the flexibility of GTF/GFF files. Despite the higher memory usage, the performance benefits are substantial, and the authors contend that these advantages justify the increased memory consumption. Future work will focus on expanding features and improving interoperability with other libraries.

The choice of C was motivated by performance considerations, and it is CPython’s native language, although programming in C has well-known drawbacks. A possible direction for future work is reimplementation in Rust, which may offer comparable performance together with stronger guarantees of safety and maintainability.

## Data Availability

No new data were generated or analysed in support of this research. Data used for benchmarking were obtained from Ensembl (Dyer *et al.* 2025). This library is available for installation from the Python Package Index (PyPI) under the name eccLib  https://pypi.org/project/eccLib/. The source code is available at https://gitlab.platinum.edu.pl/eccdna/eccLib. The version described by this document (1.1.0) is archived as https://doi.org/10.5281/zenodo.17024282. More detailed documentation can be accessed at https://gitlab-pages.platinum.edu.pl/eccdna/eccLib/. Benchmark summary with only the methods that have achieved the best result in at least one column.^a^ The best value for each metric in each benchmark is highlighted in bold.
